# Poxvirus Recombination

**DOI:** 10.3390/pathogens11080896

**Published:** 2022-08-09

**Authors:** David Hugh Evans

**Affiliations:** Department of Medical Microbiology & Immunology and Li Ka Shing Institute of Virology, The University of Alberta, Edmonton, AB T6G 2J7, Canada; devans@ualberta.ca; Tel.: +1-(780)-492-7997

**Keywords:** vaccinia virus, poxvirus, genetic recombination, marker rescue, DNA replication, DNA polymerase, E9L, strand transfer

## Abstract

Genetic recombination is used as a tool for modifying the composition of poxvirus genomes in both discovery and applied research. This review documents the history behind the development of these tools as well as what has been learned about the processes that catalyze virus recombination and the links between it and DNA replication and repair. The study of poxvirus recombination extends back to the 1930s with the discovery that one virus can reactivate another by a process later shown to generate recombinants. In the years that followed it was shown that recombinants can be produced in virus-by-virus crosses within a genus (e.g., variola-by-rabbitpox) and efforts were made to produce recombination-based genetic maps with modest success. The marker rescue mapping method proved more useful and led to methods for making genetically engineered viruses. Many further insights into the mechanism of recombination have been provided by transfection studies which have shown that this is a high-frequency process associated with hybrid DNA formation and inextricably linked to replication. The links reflect the fact that poxvirus DNA polymerases, specifically the vaccinia virus E9 enzyme, can catalyze strand transfer in in vivo and in vitro reactions dependent on the 3′-to-5′ proofreading exonuclease and enhanced by the I3 replicative single-strand DNA binding protein. These reactions have shaped the composition of virus genomes and are modulated by constraints imposed on virus–virus interactions by viral replication in cytoplasmic factories. As recombination reactions are used for replication fork assembly and repair in many biological systems, further study of these reactions may provide new insights into still poorly understood features of poxvirus DNA replication.

## 1. Biological Function of Recombination

Most of us learn about recombination in introductory genetics. There we studied the pioneering work of T.H. Morgan and his students and how that led to the discovery that inherited traits (genes) reside on chromosomes and can be mapped into linear arrays by analyzing the frequency of recombination between fruit fly genes. In subsequent years, such studies along with H. Muller’s new insights into the origins of mutations and J.B.S. Haldane’s work on population genetics were to form the foundations of what J. Huxley called the “Modern synthesis”. The modern synthesis united Darwin’s theory of natural selection with these and other new (1940s) discoveries in genetics. It served to explain how selection acting upon a population of organisms, each encoding (and sometimes expressing) different combinations of recombining traits, could drive the evolution of a species. Although Eugene Koonin has argued that “The edifice … has crumbled, apparently, beyond beyond repair” [1], for most of us, the experimental foundations of the modern synthesis still lie at the core of our understanding of genetics and genetic recombination.

From the author’s perspective, a problem with the way introductory genetics is taught is that few of us instructors have time to explain why peas and flies are having sex and recombining genes. The knowledge vacuum can lead students to adduce that recombination mostly exists to eliminate bad gene combinations and generate good ones (a solution to a problem called Muller’s ratchet [2]). However, selection only works in the here and now, it cannot anticipate the future advantages the recombination machinery might offer. Therefore, what is the purpose of these systems? The simplest answer is that genetic recombination serves an essential role in DNA repair and especially promotes replication restart at stalled or broken replication forks.

This understanding emerged in the last few decades, although the links between recombination and DNA repair had been known for many years. The *E. coli recA* gene was the first recombination gene ever found, using a screen for mutagenized strains that did not recombine in Hfr crosses [3]. Even then, it was noted that these *recA* mutants were also UV-sensitive as are many of the other recombination-defective mutants that were later identified in different organisms. These many disparate observations have been assembled into now coherent models linking replication, repair, and recombination whose development has been well-summarized in helpful reviews [4,5,6,7]. Derivatives of these models have since been proposed for eukaryotes [8,9]. Interestingly, where viruses are concerned, one can see that many potential links between repair, replication, and recombination were anticipated by Anna Skalka and Gisela Mosig in their pioneering studies of bacteriophage T4 [10], and lambda [11], replication. These studies in bacteria and phage have colored our own investigations and I will return to reconsider the implications throughout this review.

## 2. Poxviruses

Far more comprehensive descriptions of the poxvirus infection cycle can be found in many excellent reviews [12,13,14,15,16,17,18,19,20,21], and will not be reiterated here. However, if one has an interest in virus recombination then there are features of poxvirus biology deserving of special consideration. Of perhaps the greatest importance is the observation that these viruses replicate in the cytoplasm of infected cells, in compact membrane-associated structures called virosomes or factories [22]. Historically called Guarnieri bodies, each of these structures are now known to originate from a single infecting particle [23]. This mode of replication isolates one virus from a second and constrains recombination between co-infecting particles [24]. Cytoplasmic replication also restricts access to components of the cell’s DNA replication and repair machinery, while exposing poxviruses to DNA sensors that regulate innate immune responses [25]. This has likely driven the evolution of large genomes, now known from sequencing [26], and other investigations to encode many proteins required by processes like transcription and replication as well as immune evasion. Although the nucleus is clearly not an impervious barrier to enzyme recruitment (cellular DNA ligases can complement vaccinia virus (VACV) A48R mutations [27], and topoisomerase II is found in VACV factories [28]), the demonstration that virus DNA synthesis is still seen in enucleated cells [29], shows poxviruses possess a considerable degree of nuclear autonomy. The fact that all these virus replication and assembly reactions take place in the cytoplasm also provides microscopists with a clear view of these events, unobscured by nuclear components.

Collectively, these features of virus biology offer the tools and impetus to look for virus-encoded recombination activities.

## 3. Historical Insights

Poxvirus research has long been associated with smallpox research and dates back to Jenner’s pioneering discovery that one could vaccinate against smallpox using a safer agent than the variola virus (VARV) than in current use [30]. The specific virus Jenner was using remains a subject of debate, but documentary and genomic evidence collectively suggests that the modern smallpox vaccine (vaccinia virus) likely originated in horses as a horsepox virus [31,32,33,34]. In turn, these strains share ancestry with physically larger and genetically diverse families of Orthopoxviruses called cowpox viruses. While VACV remains the most intensively studied of all poxviruses, the earliest studies with some relevance to virus recombination did not use that virus. In 1936 Berry and Dedrick described how infectious myxoma virus could be recovered from rabbits injected with a mixture of heat-inactivated myxoma virus and infectious Shope (or rabbit) fibroma virus (SFV) [35]. Fenner et al. later argued that this was more likely the first illustration of poxvirus reactivation rather than genetic transformation [36], which, as discussed later, employs a helper virus to recombine and replicate the damaged DNA of a second poxvirus. Using the same methods and strains that he had used a year previously to first document poxvirus recombination [37], it was shown that reactivation reactions also generated VACV recombinants [36].

These experiments were complicated by a paucity of genetic markers and the difficulties of assaying them. For example, the phenotypes scored by Fenner and Comben in VACV-rabbitpox crosses included pocks on chorioallantoic membranes, virulence in mice, rabbit lesions, haemagglutin activity, and heat resistance. Despite the challenges, recombinants were still easily recovered leading these authors to offer the conclusion that “Recombination was not an uncommon event…” [37]. These pioneering studies were followed by others in a series of papers from the John Curtin School where recombination was used to detect and map rabbitpox “white” complementation and linkage groups [38,39], and study hybridization among and between Orthopoxviruses and Leporipoxviruses [40]. The recovery of a putative hybrid between myxoma and Shope fibroma viruses [40], anticipated the later discovery of a Leporipoxvirus called malignant rabbit virus and the demonstration that it was indeed a viable genetic hybrid [41,42].

In the absence of sequence data, the fact that these methods could be used to delineate the relationships between poxvirus strains was adapted to try and clarify the still uncertain relationships between VARV and other Orthopoxviruses. The pursuit of that goal led Bedson and Dumbell to test whether hybrid viruses could be recovered using chorioallantoic membranes as a culture environment and VARV to reactivate heat-inactivated cowpox and rabbitpox viruses. Using semi-permissive growth temperatures to modulate VARV growth, hybrids were recovered between rabbitpox and VARV minor (a.k.a. Alastrim), and between cowpox and VARV major [43,44,45]. The products of these studies were later transferred to the United States Center for Disease Control, and have reportedly been sequenced. The continued existence of these unique strains remains a matter of controversy. There have long been calls for their destruction because the knowledge gained from combining sequence, virulence and host-range data [43,44], might provide unwelcome insights into smallpox pathogenicity.

The adoption of tissue culture methods in the 1960s facilitated the production and characterization of new poxvirus mutants; these and other advances in virus biology and biochemistry were reviewed by Joklik in 1968 [46]. Recombination was being used at the time to build detailed maps of bacteriophages [47], but with poxviruses complementation and recombination analysis found a more limited use as tools for differentiating between mutants [48,49]. In an interesting study, it was shown that at least one of the two co-infecting viruses has to be replicating and that a delay in adding a second infectious virus reduced the recombination frequency (R_f_) in a time-dependent manner [50]. Although its role in recombination was still to be documented, it was also discovered that VACV infection led to the appearance of a new DNA polymerase [51,52,53] bearing a single-strand exonuclease activity [54]. In addition, advances in electron microscopy put this work into a cellular context and began to establish all the now understood events in the VACV life-cycle including the relationships between sites of cytoplasmic DNA replication and virion assembly [55,56].

The later 1960s and 1970s was an extraordinarily interesting and productive period as the molecular biology revolution swept through the field of virology. It was shown that VACV infection induced the expression of many other enzymatic activities relating to nucleic acid metabolism including a thymidine kinase [57], DNA-dependant RNA polymerase [58], DNA-dependent ATPase [59], and a DNA ligase [60]. Gene expression was regulated in a manner resembling the early and late genes of bacteriophage [61,62], by an array of proteins and regulatory elements (reviewed in [63]). The fact that some enzymes were packaged in the virion was also becoming apparent. Viruses offered tractable models for researchers who were perhaps more interested in cellular biology, but it was soon clear that poxviruses were unusually large viruses with some of the pre-restriction mapping estimates for the size of a VACV genome ranging from 190,000–260,000 bp [64,65]. These genomes are also cross-linked at the telomeres [65] and, during replication, form rapidly sedimenting, protein-coated complexes [66,67,68]. This period of transition and discovery is perhaps naturally bookmarked by the publication in 1977 of the VACV *Hin*dIII restriction map, which also hinted at what were later shown to be inverted duplications in the telomeres [69]. A legacy of these early maps is the *Hin*dIII-based naming convention that is still used to label VACV genes and proteins [26].

During this period there were also some continuing efforts to assemble and genetically map collections of temperature-sensitive and/or drug-resistant mutants [70,71,72,73]. One such study [74], used three-factor crosses and mutations later mapped to D12L and D13L [75,76], to construct a linear recombination map of the region. A molecular interpretation of these data is complicated by the fact that temperature sensitivity and rifampicin-resistant phenotypes overlap in D13L, and by the many mutations introduced by chemical mutagens [77]. However, as a first approximation one can estimate that such crosses yield ~1% recombinants per 1 kbp.

These studies were followed by more extensive efforts to generate recombination-based maps of VACV genes or genomes [78,79,80]. The experiments involved co-infecting cells with two viruses at a high multiplicity of infection, so minimizing the odds of a cell being infected with just one of the two genotypes—which cannot yield recombinants. Because of later research [75,81,82,83], one can approximate the physical location of many of the markers used in these crosses and, therefore compare the genetic and physical maps. However, as we have shown from a meta-analysis of these data [84], the classical relationship between R_f_ and physical distance is obscured by experimental noise in virus-by-virus crosses. At distances <20 kbp, these and other experiments detect ~1.5% recombinants per 1 kbp, but the variance in intra- and inter-genic crosses [83], is generally too great to use the relationships to easily and accurately assemble R_f_-based genetic maps. Linkage is lost at longer distances, but even in long-distance crosses one never recovers 50% recombinants. Since these studies incorporated the necessary two-fold correction for there being two (assumed) reciprocal classes of recombinants, it suggests that other factors operate to limit recombinant production. From a practical perspective, these problems meant that the marker-rescue technologies described in the following sections became the tool of choice for mapping VACV mutations.

The end of the 1970s also marked a significant event, the eradication of smallpox as an endemic disease [85]. As a result of the growing recognition of the hazards that this created from a public health perspective [86], Bedson and Dumbell’s studies [43,44], have never been repeated and, for the most part, VARV was abandoned as an object of discovery research.

## 4. Marker Rescue Studies

The development of methods for transfecting DNA [87], led researchers to study how this technology could be combined with reactivation methods to produce recombinant poxviruses. This was first demonstrated in three papers published in 1981 [88], and 1982 [89,90]. Properly speaking, all three papers described a process known as “marker rescue”, wherein the desired trait is encoded on a fragment of transfected virus DNA, and it can be used to generate recombinants if the transfected DNA and replicating virus DNA share regions of sequence identity (i.e., homology). Sam and Dumbell also showed that infection and transfection methods could be used to reactivate heterologous Orthopoxviruses and, if the DNA is cut, generate hybrids between rabbitpox and ectromelia virus [88]. What probably helps improve the efficiency of these reactions is that any DNA transfected into poxvirus-infected cells is also amplified through a non-specific DNA replication process that is still poorly understood from a mechanistic perspective [91]. Regardless, these discoveries opened the doors wide for constructing recombinant poxviruses of every imaginable type. For example, the method was soon used to assemble transgenic VACV recombinants encoding the herpes simplex virus thymidine kinase [92]. Readers who are interested in poxvirus vector technology and its applications can find an introduction to the topic in many informative reviews (e.g., [93,94,95,96,97,98,99]).

Since these initial discoveries, marker rescue has been widely used to map poxvirus genes (e.g., [79,80,81,100,101,102], reviewed in [103]). Perhaps the most comprehensive application of the technology is illustrated by a paper from Richard Condit’s laboratory, where it was used to map three collections of temperature sensitive VACV mutations [81]. Marker rescue remains a powerful tool for cloning dominant traits such as those encoded by an unknown genetic locus [104], and drug resistance [105,106,107]. The development of these technologies of course also sparked much interest in using recombination as a tool for genetically modifying or disrupting poxvirus genes. This has since been aided by the development of many different drug-selectable or visible markers such as VACV thymidine kinase [108], *E. coli* beta-galactosidase [109], and xanthine-guanine phosphoribosyl transferase [110], fluorescent proteins [111,112], and hybrid proteins [113]. As these investigations progressed through the 1980s, they then also began to yield new insights into the mechanism by which poxviruses catalyze homologous genetic recombination.

## 5. Recombination and Repair Models

Researchers have proposed many models of varying degrees of complexity to explain the molecular steps that yield recombinant DNA molecules. The complexity derives from the need to account for the sometimes-esoteric properties of the homologous recombination reactions that are studied in bacteria and in meiotic and mitotic eukaryotic cells. Complexity also arises when linking recombination repair to processes like replication restart and the cell cycle. Although viruses and phages may not require such schemes to recombine DNA [114], a few of these models should be described to put the terminology in context. Many excellent papers can provide a comprehensive review of this literature, two of the most accessible are reviews by Ranjha et al. [115], and Weller and Sawitzke [114]. All of these models ultimately describe ways in which a broken DNA duplex can be repaired by copying homologous sequences located on either a sister duplex (e.g., the leading and lagging strands in a replication fork), or on a second homologous duplex (e.g., transfected DNA, another genome, or chromosome).

**Figure 1:**
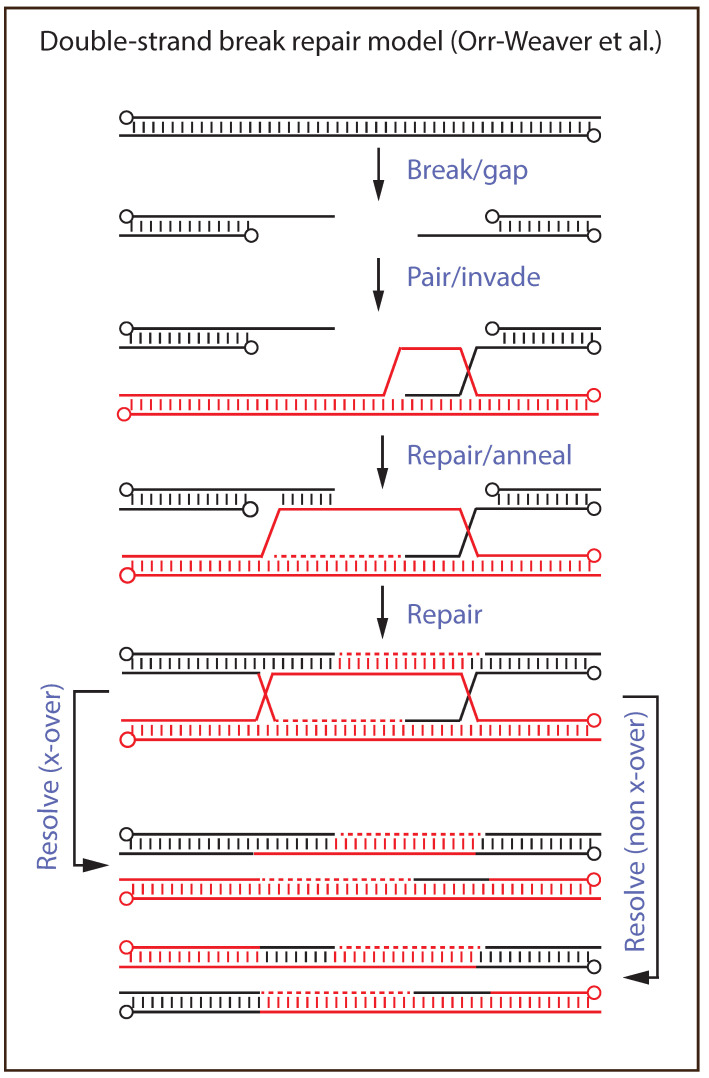
Recombination mediated by DNA strand invasion. The figure shows the double-strand break repair model for recombination and repair [118]. The first step in the repair reaction depends upon the invasion of an undamaged duplex strand by a protein-coated and homologous single-strand of DNA. Further extension of the displacement loop, combined with DNA replication and additional rounds of strand annealing and repair synthesis, serves to replace any lost sequence with DNA encoded on the intact homolog. During this process, hybrid DNA can be generated by strand exchange, which may or may not be later subjected to mismatch repair. How the two Holliday junctions are cut by a structure-specific nuclease determines whether or not the flanking genetic markers are exchanged. The 5′-ends are marked with circles.

Broadly speaking, one can categorize recombination models as either requiring the ATP-dependent invasion of a duplex strand by a single strand, through displacement (D)-loops, or simpler models dependent on single-strand annealing reactions. The models that predicted the existence of a strand invasion step led to the discovery that these reactions were catalyzed by *E. coli* RecA protein [116], as well as an abundance of orthologs in many branches of cellular life, including hRAD51 [117]. Figure 1 illustrates the double-strand break repair model [118], that in various forms lies at the heart of more embroidered recombination and repair schemes. The model provides a connection between replication and recombination and, depending upon how the Holliday junctions are resolved, can produce cross-overs (or not) in flanking markers. Because processes like this also create hybrid DNA, which may or may not be subjected to mismatch repair, they can also explain genetic phenomena historically called post-meiotic segregation and gene conversion in fungi.

**Figure 2:**
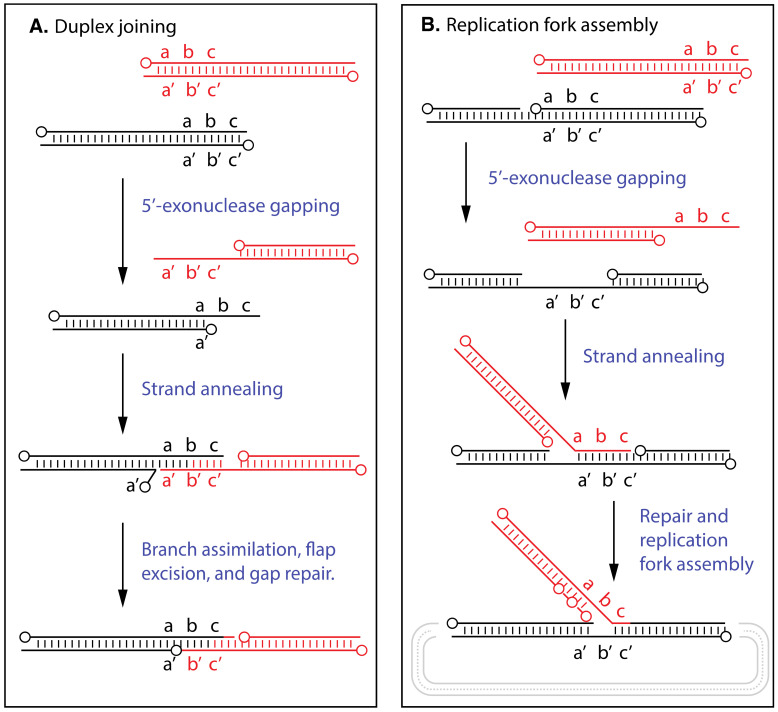
Basic elements of reactions catalyzed by two-component systems like the phage λ encoded “Red” enzymes. Panel (**A**) illustrates how the 5′-to-3′ Red exonuclease can expose varying lengths of complementary single strands, which are then free to anneal in reactions promoted by Redβ. Panel (**B**) illustrates how the process might create new replication forks if nuclease(s) has exposed complementary sequences. If the new joint is located on a circular molecule (grey oval), this reaction could initiate rolling circle DNA replication. The 5′-ends are marked with circles and the lettering (e.g., a/a’, b/b’, c/c’) indicates complementary sequences within regions of homology.

However, as I discuss below, poxvirus infections do not induce an ATP-dependent strand transferase [119], nor do poxvirus genomes encode RAD51/RecA homologs. The biochemical and genetic evidence instead suggests that poxviruses employ exonucleases and single-strand annealing reactions to produce recombinants [120]. These have been called “two-component” systems because they require just an exonuclease and a second protein to promote strand annealing. The bacteriophage λ Red (“recombination deficient”) recombination pathway, which employs a 5′-to-3′ exonuclease and Redβ “annealase”, serves as the prototype for these two-component systems [121]. The herpes simplex virus UL12 and ICP8 proteins catalyze a similar reaction [122]. Figure 2 illustrates some central elements of the reactions catalyzed by such systems, including the potential to prime formation of replication forks and rolling circle DNA synthesis. It is important to note that these pathways do not operate in isolation and there are circumstances when the actions of these primary phage or virus-encoded systems are also modulated by host enzymes. For example, both the *E. coli recA* [123], and human hRAD52 [124], pathways affect recombination of phage λ and HSV, respectively.

In contrast to homologous recombination, poxvirus *non*-homologous recombination in a much rarer process, although there is no doubt of the biological importance as horizontal gene transfer offers a route by which viruses can acquire and transmit novel traits [125]. In one example, fowlpox virus has been shown to acquire and vector an avian retrovirus [126]. Another example is illustrated by the viral Golgi anti-apoptotic protein (vGAAP), a Bax inhibitor encoded by the VACV strain Lister 196 gene [127,128]. The vGAAP gene is flanked by 21 bp duplications characteristic of LINE-1 mediated transposition events [129]. Curiously, vGAAP is not encoded by most other VACV strains, but it and some flanking genes are found in many cowpox strains and the sequence suggests an interesting history. The duplications suggest it was first acquired by transposition into an ancestral cowpox strain, but the gene more recently transferred by homologous recombination into a precursor of VACV Lister. This may not be surprising given the 19th century practise of propagating and “invigorating” smallpox vaccines (such as the Beaugency lymph [33]) by mixing different wild-sourced inocula. This process of horizontal gene transfer with target site duplications has since been replicated in culture using a selection for K3L and E3L activities [129,130]. Transfection studies have also been used to recover recombinants bearing non-homologous genes, although the viruses generated this way look different from those generated by transposition [131]. Because of the unique features of these, and perhaps related [132], kinds of events they will not be discussed further.

## 6. Transfection Studies

The observation that any DNA transfected into a poxvirus-infected cell is replicated in *trans* [91], provided a useful tool for investigating the properties of these reactions. Using Southern blots and transfected plasmids, we originally showed that the process is dependent upon infection and generates high molecular weight concatemers composed of all possible arrangements of the restriction site markers in newly replicated DNA. Importantly, these reactions required virus replication and the process could be blocked with the polymerase inhibitor, phosphonoacetic acid [133]. Similar reactions were observed in cells infected with an Orthopoxvirus (VACV) or a Leporipoxvirus (myxoma and SFV), although the SFV-catalyzed reactions were more efficient. Such high-frequency recombination reactions would cause the well-known instability of tandemly repeated poxvirus sequences (e.g., [108,134,135]) and promote indel mutagenesis [136].

A convenient feature of this process is that it generates long concatemers, and so one can transfect λ phage DNA and later convert the extracted recombinant products into phage particles by in vitro packaging. All the genetic tools devised by phage researchers can then be used to study a poxvirus problem. Using these assays, several additional features could be adduced about poxvirus (in this case SFV) recombination systems. Perhaps the most striking feature was that linkage is lost in the transfected DNAs at distances >350–500 bp. This is a high-frequency process where the 0.02% recombinants bp^−1^ is comparable to what is measured in T4 and λ crosses [137]. Even correcting for a missing class of doubly mutant recombinants, we could also never recover 50% of recombinants. This suggested that something was reducing the multiplicity of genomes interacting in what Frank Stahl has called the “mating room” [138]. Transfection methods can deposit a lot of well-mixed DNA into an infected cell, and so the limits most likely relate to how many recombining molecules can first find their way into factories where replication and recombination take place. We estimated this limit comprises 4-to-5 λ-sized DNAs [137]. Some of the crosses also exhibited a phenomenon called “high negative interference”, where many more recombinants were recovered than expected when the markers were located close together (<100 bp) or if they were arranged in Type II (i.e., repulsion) crosses.

High-negative interference is characteristic of systems that produce hybrid DNA and, in the aforementioned experiments, it is caused by *E. coli* mismatch repair systems acting on the hybrid DNA delivered by the packaged phage. These studies used pairs of λ cI mutations since the clear versus turbid plaque phenotype provided a simple assay for recombinant molecules. Interestingly, ~1% of the phage plaques showed a mottled phenotype, and the effect was enhanced 2–3-fold when the phages were plated on an *E. coli mutS* strain [139]. This offered further evidence that the molecules recombined in virus-infected cells contained significant quantities of hybrid DNA. Using denaturing gradient polyacrylamide gels, Southern blots, and plasmids encoding selected mutations, up to 25% of the mutant sites could be found transiently embedded in heteroduplex DNA in DNA recovered from SFV-infected and transfected cells. The rise and decline of the hybrid molecules correlated closely with the replication of the transfected DNAs and the appearance and disappearance of the SFV DNA polymerase activity.

## 7. Biochemical Studies

**Figure 3:**
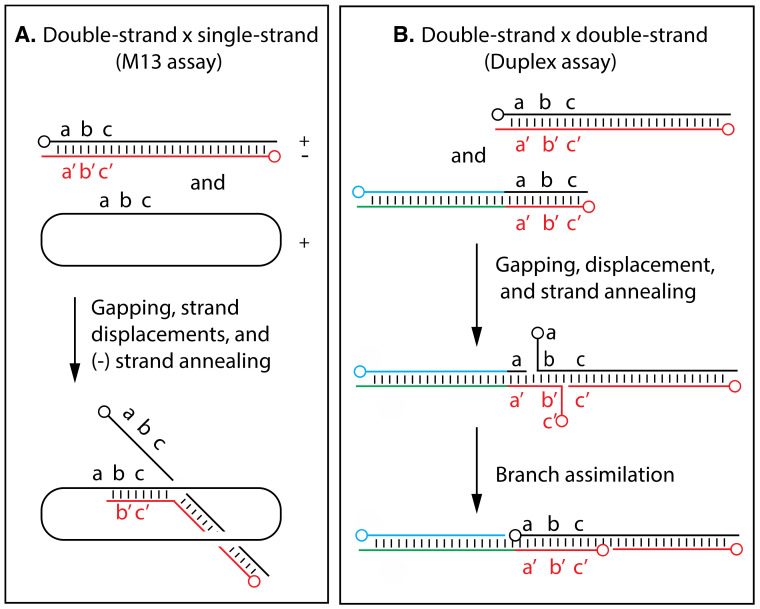
Strand transfer and duplex joining reactions catalyzed by VACV DNA polymerase. Panel (**A**) shows an assay that uses substrates comprising a single-stranded DNA circle and homologous linear DNA duplex [120]. The products bear a displaced single strand that produces an α-shaped structure in an electron microscope, and the polarity of the reaction can be adduced by incorporating blocks of non-homologous sequence on either end of the duplex (not-shown). Panel (**B**) illustrates a duplex strand joining reaction. Radioactive end-labeling showed that the products had been processed by a 3′-to-5′ exonuclease [147]. The 5′-ends are marked with circles and the lettering (e.g., a/a’, b/b’， c/c’) indicates complementary sequences within regions of homology.

These data provided the rationale for experiments designed to detect and purify an activity catalyzing the requisite strand transfer reactions. Using a gel-based assay [140,141] (Figure 3, panel A), an activity could be purified ~170-fold from VACV-infected HeLa cells that produced α-shaped joint molecules in reactions containing single-stranded M13 circular DNA and a homologous linear duplex [119]. The reaction was ATP independent and by locating non-homologous blocks of sequence on the ends of the linear duplex molecule, one can deduce that the hybrid DNA is formed and extended in a 5′-to-3′ direction relative to the complementary target sequences located on the (+) stranded single-strand circle. The purest fractions contained DNA polymerase activity and three proteins (approximately 110, 52, and 32 kDa) that later research [142], suggests are likely the heterotrimeric replication complex comprising E9, A20, and D4 with sizes of 117, 49, and 25 kDa, respectively. However, at the time the imperfect chromatographic correlation between polymerase and recombinase activity created doubts that the polymerase was the strand transferase [119].

To clarify the situation we used a method devised by McDonald and Traktman [143], to prepare a highly purified form of monomeric VACV E9 polymerase and showed that it too catalyzed strand transfer reactions [120]. This study also showed that the reaction was stimulated by adding VACV I3 single-strand DNA-binding (SSB) protein [144,145,146], and transfection and biochemical studies showed that a C*ts42* E9L mutation destabilized the recombinase activity in vivo and in vitro. With this highly purified form of VACV DNA polymerase, one can show that the enzyme also catalyzes another reaction (Figure 3, panel B) that joins together two (or more) linear DNA duplexes bearing overlapping end homology [147]. Radioactive end labeling showed that the duplex–duplex joining reaction was dependent upon limited attack on the molecules by the E9 3′-proofreading exonuclease.

**Figure 4:**
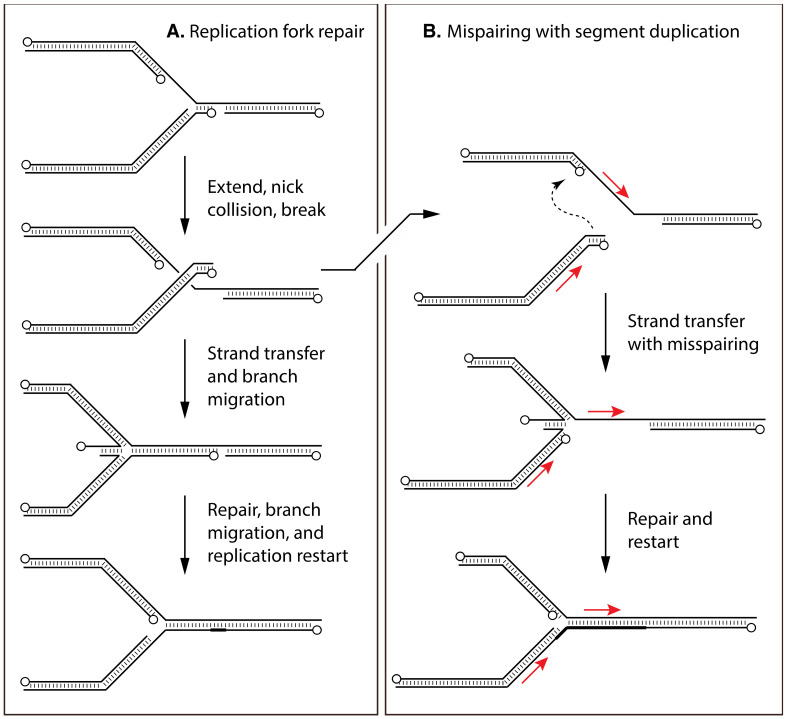
Hypothetical ways in which a two-component recombination reaction can repair broken replication forks. Panel (**A**) shows how a migrating replication fork is broken through collision with a nick. Fork reversal combined with strand transfer, annealing, and branch migration can reconstruct the broken molecule. This provides time for repair and restart. The “chicken foot” is a Holliday junction, and could be processed in various ways by enzymes such as A22 and/or G5. Panel (**B**) illustrates how mispairing could produce an aberrant joint and duplicate adjacent sequences (red arrows). The 5′-ends of are marked with circles.

The simplest model that can ties together these studies would be if the 3′-end of the duplex (-) strand was transiently attacked by the 3′-5′ exonuclease and then additional portions of that 3′-ended strand were displaced by the enzyme tracking in a 5′-to-3′ manner relative to the duplex (+) strand. The displaced 3′-ended strand would then be free to hybridize with either the (+) stranded single-strand circle (in the M13 assay) or with sequences exposed by another enzyme attacking a second homologous duplex (in the duplex–duplex assay) (Figure 3). A critically important feature is that these reactions offer a possible route by which broken replication forks can be reassembled and replication restarted through replication fork reversal [148], and likely reflects the biological driver behind the evolution of such a system (Figure 4, panel A). These reactions also produce the 3′-ended primers that can prime more DNA replication and, like λ Red recombination, could prime a rolling hairpin process that produces viral concatemers. Subsequent studies identified some unique reaction features that differentiate E9 from other proof-reading polymerases. These include the fact that E9 has an unusually high affinity for duplex ends, can process molecules containing single-stranded 3′-ended internal branches into structures containing meta-stable and ligatable nicks, and that physiological dNTP concentrations are not profoundly inhibitory [149,150].

These I3·E9 catalyzed single-strand annealing reactions clearly belong to the two-component category of recombination schemes while differing from systems like λ Red in that the poxvirus reaction is initiated by a 3′-rather than 5′-exonuclease. The model is unusual and proving that poxvirus recombination employs such a scheme in vivo presents a challenge. A polymerase is required, but is that only because it is needed to replicate the molecules that are being recombined and analyzed? The 3′-5′ exonuclease is special in one sense in that unlike other proofreading exonucleases (e.g., T4 DNApol [151]) the VACV E9L nuclease is an essential function [152]. Perhaps the best in vivo evidence in support of the hypothesis comes from studying the behavior of recombination substrates where the 3′-ends have been modified by incorporating molecules of the antiviral drug, cidofovir (CDV). CDV inhibits the activity of both the E9 3′-5′ exonuclease and the 5′-3′ polymerase [153], but these effects are suppressed by an E9L^A314T^ drug-resistant mutation in the exonuclease domain, which enhances excision of newly incorporated CDV molecules [152]. Using both biochemical and transfection studies, one can show that filling CDV into the ends of the DNA inhibits E9-catalyzed duplex–duplex joining reactions in vivo and in vitro, but this inhibition is overcome if one transfects CDV-blocked substrates into cells infected with an E9L^A314T^-mutant virus. Many other of the reaction features observed in vitro were subsequently documented in vivo using plasmids as substrates in transfection assays [154,155]. This included the observation that linearized DNAs recombined much more efficiently than circular substrates and that as few as 12–16 bp of overlapping homology suffice to support VACV-catalyzed duplex–duplex recombination. The reactions have some tolerance for mismatched base pairs and so, for example, a C·A mismatch with a C in the 3′-ended and an A in the 5′-ended strands can be used to track the fate of each strand near that end. After transfecting these DNAs and retrieving the recombinant plasmids, sequencing showed that the nucleotide near the 5′-end (the A in this example) was four times more commonly recovered in the mature junctions than the C in the adjacent 3′-end [155]. Collectively, these studies all implicate the VACV E9 3′-5′ proofreading exonuclease as an important driver of single-strand annealing reactions in vivo.

Although a strand-transfer step is needed to catalyze the initial formation of recombination intermediates, other gene products would still be required to process the joint molecules into mature recombinants (Table 1). For example, the “chicken-foot” structure [148] seen in Figure 4 (panel A) is topologically a Holliday junction and such branched structures, even if not further replicated, still need to be disarticulated to permit packaging. A need for a Holliday junction resolvase was also long ago proposed to play a role in converting concatemeric replication intermediates into hairpin-ended genomes [156]. The VACV A22R gene was later discovered to encode a homolog of the RuvC family of bacterial Holliday junction resolvases and A22 shown to cleave Holliday and other branched DNA structures [157,158]. The observation that A22R is a late gene supports the hypothesis that it is more likely required to debranch or decatenate the DNA in advance of packaging. Bioinformatics also led Upton and colleagues to propose that VACV G5R encoded a FEN-1 like flap endonuclease [159]. These enzymes cleave unpaired strands from duplex substrates. Subsequent studies showed that, while G5R is not entirely essential (perhaps because the E9 exonuclease can also catalyze FEN-1 like reactions [149]), a VACV ΔG5R mutant exhibited a phenotype consistent with Upton’s proposal. Most notably, it produced fragmented DNA, packaged DNA improperly, and exhibited a defect in double-strand break repair [160]. Although poxvirus Type I topoisomerases can catalyze site-specific strand transfers between molecules encoding a 5′-(C/T)CCTT-3′ motif [161,162,163], its primary role most likely still resides in the topoisomerase function as the presence of the motif and its variants is not associated with any unusual increase in the frequency of homologous recombination [164]. While it is difficult to exclude the possibility that there are still other cellular and viral enzymes involved in catalyzing VACV recombination, the D5 ATPase/helicase being a possible candidate, at first glance the virus appears to encode all the essential proteins (E9, I3, A22, G5, and A48 (DNA ligase)) that would be needed to collectively catalyze these reactions.

## 8. Sequencing and Genomics

The application of sequencing and genomics technologies has provided further molecular insights into how recombination affects poxvirus biology. An early example is illustrated by the sequencing of portions of the malignant rabbit virus, which showed that it was a natural hybrid [42]. The virus likely arose when DNA encoding a portion of the SFV left ITR and ITR-junction replaced homologous sequences encoding the myxoma virus right ITR and its junction, and then the new genome rearranged again to extend and mirror [173], the sequences encoding the recombinant ITRs. More recently a novel strain of lumpy skin disease virus (LSDV) was discovered in Russia [174]. The LSDV Saratov (2017) strain appears to have been formed by recombination between field and vaccine strains, something Gershon and his colleagues had predicted many years ago given the close similarities between Capripoxviruses [175,176]. Bioinformatics [177], can also be used to detect old past episodes of recombination by applying phylogenetic analysis to different segments of virus genomes. For example, using these methods it was shown that the VARV minor strain BRZ66_39 encodes blocks of sequence in the left and right ends that bear a disproportionate resemblance to homologous sequences in the VARV major strain NIG69_001 [178]. Similarly, the “patchy” pattern of polymorphic alleles found in Dryvax vaccine strains suggests that these swarms of non-clonal viruses have been exposed to repeated rounds of recombination [179]. One such ~150 nt patch in the I4L gene of a Dryvax clone encoded a frameshift mutation and flanking polymorphic sites more similar to a horsepox virus I4L locus than to other VACV I4L genes in these stocks. This may be a “molecular fossil” reflecting the early history of North American smallpox vaccines [33,180,181].

**Figure 5:**
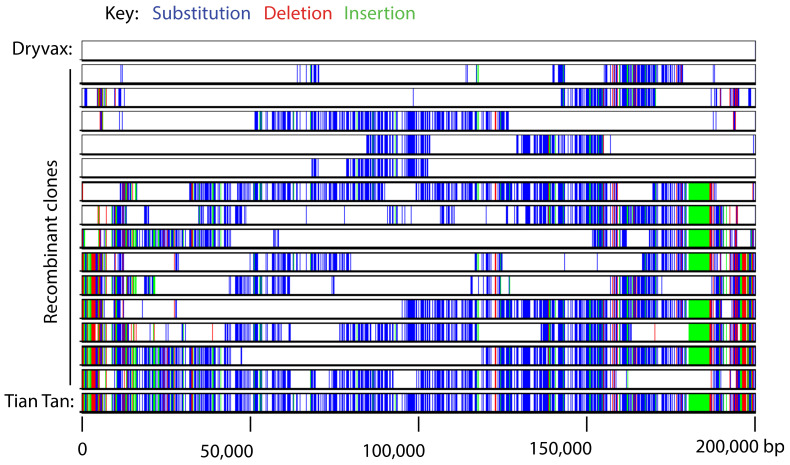
Patchy pattern of recombination between two strains of VACV [182]. Cells were co-infected with two viruses cloned from stocks of Dryvax and Tian Tan vaccines, harvested 24 h later, replated, and random progeny picked, plaque purified, and sequenced. The figure was constructed using Base-by-Base [183], and maps sites where the Dryvax strain (top row) differs from the Tian Tan strain (bottom row). These sites are distributed in patches of different length among the virus clones randomly retrieved from the co-infection.

The polymorphic sites (SNP’s) that have accumulated during passage and through geographical isolation of smallpox vaccines provide a tool for exploring how much recombination any given genome has been exposed to. This is illustrated by an experiment in which cells were co-infected with strains cloned from North American Dryvax and Chinese TianTan vaccines [182]. These genomes differ by ~1400 single-nucleotide polymorphisms (SNPs), roughly one per 140 bp. After one or five rounds of passage, the progeny were randomly cloned, sequenced, and the SNP patterns used to identify which parent a segment derived from. The recombinants again exhibited a patchy pattern composed of blocks of sequence derived from one or the other parent. A single round of cell passage comprises at least 2^14^ doublings (or perhaps more as only a portion of the DNA is packaged into the infectious particles measured in plaque assays) and was associated with at least 18 ± 11 exchanges per genome (Figure 5). Bearing in mind that the viruses retrieved at the end of the first passage bear the imprint of all the preceding rounds of replication and recombination, 14 doublings and 18 ± 11 exchanges per genome is not incompatible with every genome undergoing one recombination event per replication cycle. There is a notable discrepancy in that the 1.5% recombinants per 1 kb calculated from genetic considerations equals 100% recombinants per 66 kb or ~3 exchanges per genome. This is much less than 18 ± 11 determined by sequencing. The most likely reason is that the viruses selected for sequence analysis were screened to identify the subset bearing recombinant ends and so avoid sequencing uninformative genomes. To the extent that a portion of viruses might have replicated without yielding detectable recombinants in both studies, they would have been counted in genetic studies as non-recombinants, while the sequencing studies characterized only recombinants. The reasons for why some genomes would not have been recombined at all are discussed below.

These experiments do also illustrate a still poorly understood feature of poxvirus recombination, regarding how much sequence drift it takes to genetically isolate one family or strain of viruses from another. One SNP per ~140 bp is of little consequence as illustrated by the preceding study. At the other end of the spectrum, hybrids are never recovered when SFV is used to reactivate VACV, where even the most highly conserved gene homologs (e.g., S068R and J6R) are only ~75% identical [184]. Plasmid transfection studies have shown that two patches of flanking homology of <20 bp can still yield a few recombinants if the DNA is linearized, but practically speaking >50–100 bp of flanking homology are still required to produce recombinants in abundance. How a few embedded mismatches might further affect the efficiency of these reactions has not been studied in detail. From a practical perspective it may be that what is defined as a “genus” in poxvirus taxonomies (e.g., Leporipoxvirus versus Orthopoxvirus) is partly due to genetic isolation through sequence drift, but further confounded by factors like host range and intergenic incompatibilities.

Sequencing has also provided insights into another related genetic phenomenon involving gene amplification under selection for increased gene expression. This was first discovered by Mathews and colleagues while studying the VACV F4·I4 ribonucleotide reductase complex. VACV ribonucleotide reductase is inhibited by hydroxyurea, but continued exposure to the drug selects for resistant mutants. Many of the drug-resistant viruses encoded multiple copies of the F4L gene arrayed as a tandem repeat and flanked by novel joints [134]. The mechanism likely involves the creation of a direct duplication of a segment of DNA through non-homologous recombination, followed by the amplification of the repeat by homologous recombination. Such structures are unstable and revert to the single copy state absent selection. A similar effect was later seen using a selection for resistance to protein kinase R [185]. This requires the VACV K3L gene and, under conditions where K3 is poorly able to suppress the kinase’s activity, an array of up to 16 K3L genes are formed, acquires and homogenizes any adaptive mutations, and then collapses to leave behind a single copy of K3L again [186]. Importantly, Elde’s accordion model provides a route around the “you can’t get there from here” problem, permitting a screen for new adaptive mutations while polyploidy preserves an essential gene function. Poxvirus genomes encode many examples of adjacent paralogous genes [187]. For example, the two mRNA decapping enzymes encoded by the VACV D9R and D10R genes may well have arisen via this pathway.

It is conceivable that these tandem duplications are a common but ephemeral feature of poxvirus genomes. One possible explanation for their origin arises from the hypothesis that the primary purpose of the E9-catalyzed reactions might be to promote repair of broken replication forks [150]. Under normal circumstances, the sister strands and homologous recombination can be used to reassemble a structure that was broken when the replication fork collided with a nicked template (Figure 4, panel A). However, if the strands were first accidentally joined through a mispairing event (a reaction that would be promoted by hydroxyurea inhibiting dNTP synthesis), then repaired and replicated, it would duplicate a segment of sequence separated by a novel joint (Figure 4, panel B). Thereafter, the new duplication creates an opportunity for further rounds of inter- or intra-molecular recombination between the duplications [186], to expand or contract the copy number.

## 9. How Does the Cellular Environment Influence These Events?

A characteristic feature of poxvirus-catalyzed recombination reactions is that one never recovers equal numbers of parental and recombinant viruses (i.e., 50% recombinants), even if the markers are located far enough apart to lose the genetic linkage. This is a classical property of phage and virus systems where various phenomena conspire to reduce the maximum recombination frequency below the 50% seen in classical meiosis. Broadly it reflects the fact that some of the input genomes are replicating in isolation from others, for various reasons, and so can yield only progeny of the input parental (P) class. Isolated viruses can, of course, still recombine with replicating duplicates of themselves, but these “invisible” interactions cannot yield genetic recombinants (R). Since the recombinant frequency (R_f_) is calculated using the formula R_f_ = 100% × N_R_/(N_R_ + N_P_), where N equals the numbers of parental or recombinant viruses, any extra contribution of viruses to the N_p_ class ensures R_f_ will be less than 50%.

This situation was recognized long ago by bacteriophage researchers [138], and it reflects the combined effects of random chance affecting how many genomes enter a cell and the relative proportion of each of the two recombining viruses. Thereafter, the yield of recombinant viruses is further affected by the many different possible “lines of descent” (i.e., who recombines with who and how many times), replication and packaging kinetics, and the physical constraints on mixing imposed by the cell. Insofar as poxviruses are concerned, the fact that each infecting genome is initially sequestered within a separate cytoplasmic factory can well be expected to affect the frequency of recombination between coinfecting particles.

The cytoplasm offers a window where DNA–DNA interactions can be followed using fluorescence microscopy and one can visualize dynamic phenomena once only inferred from genetic modeling. DNA-specific dyes often interfere with virus replication and so are not well-suited to live-cell imaging. However, fluorescent DNA-binding proteins generally cause less severe problems from this perspective, and can be constructed by fusing λ Cro DNA-binding protein to fluorescent molecules like enhanced green fluorescent protein (EGFP) or mCherry [188]. These proteins can be encoded by the cell [84], or by the virus [24,189], and in either case fluorescence can be used to tag and track the sites of virus DNA replication and recombination. Collectively, a number of interesting features of poxvirus recombination reactions, and the links to virus biology, can be adduced using this technology.

First and foremost, these methods illustrate the impact that the multiplicity of infection (MOI) has on the likelihood that VACV recombinants might be formed. Of course, MOI will affect the likelihood that a cell gets co-infected with two different parental strains in the first place, but it also affects the likelihood that at some point in the infection two different factories will collide, fuse, and mix their viroplasm. For example, in cells initially infected with five factories only 2–3 collisions and fusions are observed in the first 4 h of the infection [84]. As the number of co-infecting particles is reduced, the time to collision also increases, so that a first collision is detected within 20 min in cells bearing ~10 starting factories, but this takes more than an hour, on average, in cells bearing only two virosomes. The effect of these delays on DNA mixing can be further analyzed using fluorescence in situ hybridization (FISH) to examine the location, within the intersecting factories, of DNA sequences that can differentiate between the two parents. As the time increases between the first appearance of virosome and a fusion event, the less-well mixed are the two genotypes judging by the overlap between the two FISH signals [84]. Correlative light and electron microscopy detects an abundance of cytoplasmic constituents separating the factories, including mitochondria and intracellular membranes, that perhaps present impediments to DNA mixing [189]. Collectively, this combination of factors (only half the factories collide and mix and only half these pairings can yield a recombinant, the collisions are often delayed, and viroplasm mixes poorly) all conspire to decrease N_R_ relative to N_P_ and thus decrease R_f_. Because all these parameters are stochastic in nature, they would also combine to create greater experimental variation. This effect can well explain the difficulties encountered trying to accurately map poxvirus genes using classical genetic crosses to measure recombination frequencies.

**Figure 6:**
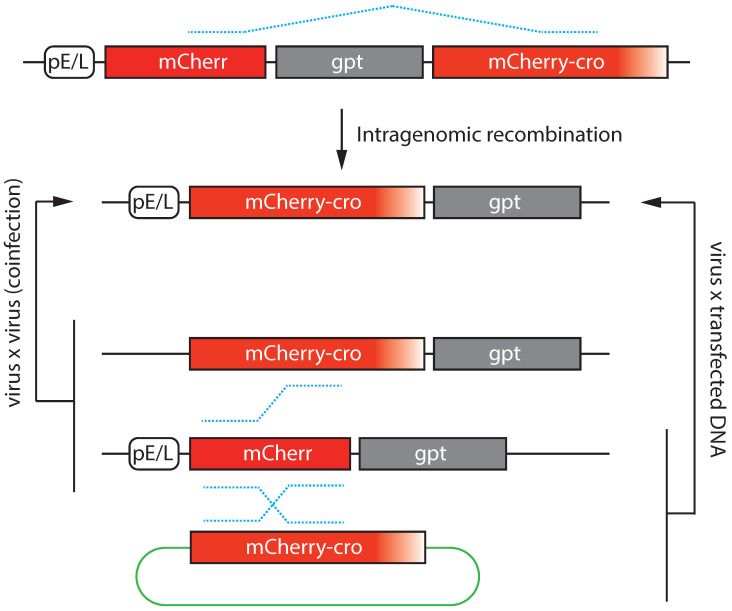
Monitoring recombination kinetics using genes encoding fluorescent proteins [24]. The figure illustrates the different ways that genes and gene fragments can be distributed to track VACV genetic recombination (dotted blue lines). Intragenomic recombination can be monitored using a split gene construct encoding an mCherry-cro DNA binding protein driven by an early-late promoter (top diagram). This arrangement is unstable and can only be maintained by selection for the interdigitated xanthine–guanine phosphoribosyl transferase (gpt) gene. Recombination between two co-infecting viruses can be monitored by splitting the mCherry elements between two different viruses. One encodes an incomplete fragment of the mCherry-cro protein plus promoter (“pE/L-mCherr”) and the other encodes a complete copy of the mCherry-cro protein but lacks a viral promoter (“mCherry-cro”). Recombination between a virus and transfected DNA (green oval) can similarly be assayed by encoding the promoterless mCherry open reading frame on the transfected DNA. In this case recombination would also partially duplicate the mCherry gene and incorporate plasmid sequences as well (not entirely shown).

These methods also illustrate something regarding the kinetics of recombinant gene formation [24]. This can be done by splitting an early-late (E/L) regulated gene, encoding an mCherry-cro reporter protein, into two overlapping fragments that can be recombined to reconstruct the gene and create a fluorescent signal. Depending upon how the fragments are arranged, the method can be used to detect inter- or intra-viral recombination as well as recombination between the virus and transfected DNA (Figure 6). At the same time the growth and movement of the factories can be tracked using cell-encoded EGFP-cro protein. These studies showed that when a single infecting virus genome encoded both mCherry-cro fragments separated by a selectable marker, it exhibited two patterns of gene expression. In some cells the protein is detected immediately and this first class derives from the recombinant mCherry-cro genes initially present in the virus stocks, while in other cells the red fluorescence shows up in abundance ~3.3 h after the factories first appear and at a time when late gene expression starts. Thus, the second class of recombinants were formed after the early promoter element was silenced and before the late-promoter element was activated, presumably during DNA replication. Recombination between VACV and transfected DNA exhibits the same kinetics, with recombinants also being formed in the period prior to the onset of late gene expression. In contrast, one does not begin to detect low levels of mCherry fluorescence until ~5.1 h after the factories appeared in cells co-infected with viruses bearing gene fragments arranged in *trans*. These studies recapitulate what is clear from genetic analysis. Intragenomic recombination is a far more rapid and efficient process than what is seen in virus-by-virus crosses.

From these studies one can take away several conclusions. First, there are several impediments which collectively restrict recombination between co-infecting poxviruses. These can be detected when one examines virosome dynamics and the timing of recombinant formation. Secondly, because co-transfected DNAs are delivered in a premixed format into poxvirus factories [190], these DNAs can be replicated and recombined by the virus machinery, while avoiding the timing and mixing challenges that beset co-infecting viruses. Thus, although the underlying reactions are likely the same, virus biology creates a bias where transfected DNA has the potential to be recombined more frequently in transfected cells than is virus DNA in virus-by-virus crosses. Finally, replication is intimately linked to recombination but by the time co-infecting viruses are starting to mix and recombine, replication is ending and mature viruses (MV) are being formed [189]. These packaging reactions will ultimately preclude the formation of any more recombinant viruses. Thus, as John Cairns predicted more than 60 years ago [23], the fact that poxviruses replicate in factories has a profound impact on virus genetics and the yield of recombinants.

## 10. Applications and Implications

Recombination reactions provide a well-established tool for genetically modifying poxviruses and are widely used to mutate virus-encoded genes or introduce new ones. The products find uses in discovery research and as vaccines and, more recently, as experimental oncolytics for treating cancers. Many improvements on the technology were devised in the years subsequent to the original descriptions of marker rescue [88,89,90]. Some applications take advantage of the fact that poxviruses can vector large inserts [191], and this permits delivery of multiple antigens such as the three Ebola glycoproteins plus a nucleoprotein expressed by MVA-BN-Filo [192]. A method that is worthy of special note is called “*trans*-dominant selection” which employs a knock-in, select, and excise-out strategy to produce marker-free recombinants [193,194]. It has a great advantage in that the resolution step should yield both parental and recombinant progeny, but recombinants are not recovered if the mutation disrupts an essential gene. More recently CRISPR technology has been used to target transfected DNA fragments to breaks in replicating viruses [195], and to enhance the efficiency of recovering recombinants [196].

Another interesting application of these technologies exploits the duplex-to-duplex joining reaction catalyzed by VACV DNA polymerase (Figure 3, panel B). The reaction is simple to set up and very efficient, and can be used to join linearized vectors to DNAs amplified with PCR primers bearing 15–20 bp of added vector end homology. The resulting joint molecules are stable enough to transform bacteria and the method has found commercial utility in InFusion cloning kits [197,198], which, when first sold, employed E9 polymerase. It is a curious coincidence that two established commercial recombineering systems were devised using VACV enzymes, the other being Topo cloning kits which use the strand joining reactions that Stuart Shuman showed [161], are catalyzed by VACV topoisomerase I.

Reactivation and recombination reactions have also found several applications, principally as a tool for more efficiently genetically modifying these viruses. One early example used λ Red recombination in *E. coli* to engineer a bacmid encoding a full-length copy of VACV, and then used fowlpox to rescue the clone as infectious virus [199]. The utility of the method was later well illustrated by constructing a bacmid encoding a copy of chorioallantois VACV Ankara, sequentially introducing six large deletions into the cloned genome in bacteria, and then testing each of the reactivated variants to see if the mutant clone(s) would duplicate the host range defects characteristic of modified VACV Ankara (MVA) [200], (they did not and only later was the defect traced to the C16L/B22R gene [201]). Recombinant viruses can also be assembled using reactivation methods and requiring repair of double-stranded breaks. For example, SFV can reassemble and reactivate VACV from mixtures of restriction fragments as long as no more than 8–10 exchanges are required to yield a contiguous genome [202]. An SFV helper virus can also be used to reactivate recombinants with near quantitative yields, if the transfected genome is first cut at restriction site(s) spanned by a co-transfected repair fragment bearing homologous ends [202]. CRISPR technology can be used the same way but offers far greater control over the cut site(s) [203].

Finally, reactivation reactions have also been used to reconstruct and reactivate poxviruses using DNA fragments obtained through wholly synthetic approaches. The first such example was a 220 kbp horsepox virus [204], but synthetic copies of VACV strains MVA [205], and Acambis 2000 [206], have since also been constructed in similar ways. An advantage of these methods is the flexibility that gene synthesis offers, and as the cost of gene synthesis continues to decline [207], and interest in more complex and personalized recombinants grows, may displace many of the methods devised to date. It makes possible the construction of mutants that would be difficult to produce in any other way [206]. Of course, the technology is not without controversy. Many countries have long restricted possession of bacterial clones encoding extensive portions of the VARV genome, because of concerns that a complete set of the larger clones could perhaps be used to reactivate VARV. The advent of cheap gene synthesis renders this check obsolete and now places a responsibility on the suppliers and consumers to cooperate on policing what DNAs can and cannot be obtained this way.

## 11. Unanswered Questions and Way(s) Forward

There remain many opportunities for further research into the biology behind poxvirus recombination systems. The most obvious questions concern the relationship between replication and recombination, although, that is greatly complicated by lingering uncertainty about the mechanism of poxvirus replication itself and by the fact that the two processes seem to be inextricably interlinked. A replication-proficient but recombination-deficient mutant has never been identified through a mutant screen [208], or by a concerted effort to mutate an obvious target like the proofreading exonuclease [152]. Poxvirus replication was originally speculated to employ a parvovirus-like continuous model for replication, with DNA synthesis initiated from a nick in a hairpin telomere followed by strand displacement [209,210,211]. However, the discovery that the D5 ATPase-helicase exhibits primase activity [169], supports a more complex, but arguably more mainstream model, involving discontinuous DNA synthesis at replication forks (e.g., [168]). The two models are not incompatible in that the (Pogo–Moyer–Graves scheme can provide a way to initiate replication, followed by a switch to a D5-dependent discontinuous mode of synthesis. However, all of this still needs to be proven.

If this hypothesis *is* basically correct, then it remains to be tested whether the strand transfer reactions catalyzed by enzymes like VACV E9 play any role in this process. In particular, can they provide a way to assemble or repair the broken replication forks that would inevitably form when a virus tries to replicate a 150–220 kbp genome? Without some such repair capacity it would be difficult to explain how poxviruses can exhibit particle-to-PFU ratios approaching 1:1 [206] and a minimal infectious dose that may be as little as one infectious unit for myxoma virus [212]. Similar strand-priming might also explain how any DNA can be replicated and assembled into concatemers in transfected cells [91], absent any clearly established origin of replication beyond the stimulatory effect of the telomeres [213]. Such links between replication, recombination, and fork repair have been explored in yeast and human cells using technologies like Tus/*Ter* traps [214,215,216]. It would be of great interest to test whether the same approach could be used to block, stabilize, and retrieve migrating VACV replication forks.

There also remain unanswered questions regarding how poxvirus DNA polymerases can catalyze strand transfer reactions. A random selection of commercially available B-family polymerases (e.g., T4 DNA polymerase) will not catalyze the same reactions. The evidence suggests that E9 catalyzes only a limited attack on 3′-ended duplex substrates, and can then melt enough of the remaining duplex end to promote strand annealing if a complementary sequence is present. I3 stimulates the strand transfer reaction, perhaps by transiently trapping greater quantities of exposed single strands and then favouring a search for homology. One clue to how all of this might be accomplished is suggested by the presence of an unusually long β-hairpin “finger” within the 3′-5′ exonuclease domain [171]. This element comprises E9 resides 299–319, and it is also where a key cidofovir-resistant mutation, A314T, is mapped [107]. This structural feature of replicative DNA polymerases is thought to play a role in switching the enzyme between editing and elongation modes, and in promoting the separation of primer and template strands, while holding onto the DNA and maintaining the processivity of the enzyme [217]. Given that VACV employs A20 as a processivity factor, it is possible that E9 polymerase has further adapted the β-hairpin to enhance the strand-separation reaction and thus favour strand transfer and I3-promoted annealing reactions. Mutational analysis of the β-hairpin element may begin to provide an explanation for E9′s curious catalytic properties.

Ultimately any such investigations should be complemented with efforts to assemble an in vitro replication system catalyzed by highly purified proteins. Although it is likely that the polymerase preparation that we first used to demonstrate strand transfer activity was purified in a form complexed with A20 and D4 [119], better-defined combinations of pure protein components are needed to answer these questions and extend the models. This could provide insights into how the activities of wildtype and mutant E9 proteins are modified when complexed with A20 and D4 and, ultimately, how these reactions are modulated by ancillary proteins like I3, the H5 scaffolding protein [16,21], and D5 helicase [218]. 

## Figures and Tables

**Table 1:** VACV proteins with links to replication and recombination.

Protein	Gene	Biochemical Activity	Role in Recombination	Reference
DNA polymerase	E9L	3′-5′ exonuclease 5′-3′ polymerase	Strand transferase	[16,147,152]
Single-strand DNA binding protein	I3L	Replicative high affinity SSB	Stimulates E9-catalyzed strand transfer	[144,145,147]
RuvC-like Holliday junction resolvase	A22R	Branch-specific endonuclease	Cleaves 3- and 4-branched DNAs	[157,158]
FEN1-like flap endonuclease	G5R	Single-strand endonuclease	Cleaves single-stranded DNA flaps	[159,160]
DNA ligase	A48R	DNA ligase, binds cell topoisomerase II	Repairs nicks	[28,165]
ATPase-helicase and primase	D5R	Helicase (putative), uncoating, primase	(hypothetical)	[166,167,168,169]
Uracil glycosylase	D4R	Uracil glycosylase, E9 processivity component	(hypothetical)	[16,142,170,171]
A20	A20R	E9 processivity factor	(hypothetical)	[16,171,172]

## Data Availability

Not applicable.
